# Antibiotic resistance and associated resistance determinants in different *Salmonella enterica* serovars isolated from pigs in Argentina

**DOI:** 10.14202/vetworld.2022.1215-1220

**Published:** 2022-05-20

**Authors:** Julián Parada, Marcelo Galas, Diego Faccone, Pablo Tamiozzo, Alicia Carranza

**Affiliations:** 1 Department of Animal Pathology, Facultad de Agronomía y Veterinaria, Universidad Nacional de Río Cuarto, Río Cuarto, Córdoba, Argentina; 2 Consejo Nacional de Investigaciones Científicas y Técnicas (CONICET), Argentina; 3 Servicio Antimicrobianos, National and Regional Reference Laboratory in Antimicrobial Resistance, Instituto Nacional de Enfermedades Infecciosas (INEI)-ANLIS “Dr. C. Malbran,” Buenos Aires, Argentina; 4 Antimicrobial Resistance Special Program, Communicable Diseases and Environmental Determinants of Health, Pan American Health Organization, Washington, DC, United States of America

**Keywords:** environment, health, livestock, salmonellosis

## Abstract

**Background and Aim:**

Salmonellosis is one of the most common foodborne diseases in the world, and the increasing antibiotic resistance in *Salmonella enterica* subsp. *enterica* recovered from food animals constitutes an important risk from a One Health approach. This study aimed to characterize antibiotic resistance and some of its associated resistance determinants in different *S*. *enterica* serovars isolated from pigs in Argentina.

**Materials and Methods:**

A retrospective study was conducted on *Salmonella* strains isolated between 2011 and 2015 from pigs in the Pampean region of Argentina. The antimicrobial susceptibility patterns to 21 antimicrobials and some antibiotic resistance determinants were characterized in 55 *Salmonella* isolates, representing 58 farms.

**Results:**

We identified 56% (n=30) of the strains as multidrug-resistant, where resistance to tetracycline (62%, n=34), ampicillin (53%, n=29), nalidixic acid (53%, n=29), chloramphenicol (33%, n=18), and trimethoprim-sulfamethoxazole (31%, n=17) was most common. The wide range of resistance to ampicillin correlates with the presence of TEM type β-lactamases in the strains. However, high susceptibility was found in the new generation of β-lactams. Fluoroquinolone resistance is a major concern. Most strains with decreased susceptibility to ciprofloxacin showed *gyrA* mutations and plasmid-mediated quinolone resistance gene *qnrB*.

**Conclusion:**

Here, we identified broad resistance to some antibiotics frequently used in human therapeutics and several easily transferable resistance mechanisms that could endanger public health.

## Introduction

Salmonellosis is one of the two most common foodborne diseases in the world. In the European Union alone, 91,857 cases of non-typhoidal salmonellosis were reported in humans in 2018; almost one in three cases could be attributed to the consumption of fresh pork products [1]. The previous studies found that approximately 7-8% of the pork at retail markets in Argentina is contaminated with *Salmonella* spp. [2,3], but few studies have characterized this pathogen in local pig production farms [4,5]. Both pathogenic and non-pathogenic bacteria have become increasingly resistant to antibiotics in recent years because of increased administration of antibiotics, especially prophylactic in-feed antibiotics, in animal agriculture [6]. The European Antimicrobial Resistance Surveillance Network recently reported increasingly prevalent *Salmonella* strains resistant to antibiotics such as quinolones, ampicillin, sulfonamides, and tetracyclines [7]. Increased antibiotic-resistant strains of *Salmonella enterica* in food animals endanger public health. Epidemiological knowledge about zoonotic pathogens and their antibiotic resistance is fundamental to a One Health approach.

Antibiotic resistance occurs when bacteria undergo mutations or acquire genes through horizontal transfer, where integrons carrying resistance gene cassettes are considered the most common forms of mobile gene element transfer. Typically, Gram-negative bacteria produce β-lactamases, such as TEM enzymes, that hydrolyze drugs, such as penicillin and older cephalosporins, resulting in resistance to β-lactam antibiotics [8]. Fluoroquinolone resistance is associated with specific point mutations within the quinolone resistance-determining regions (QRDRs) of DNA gyrase (*gyrA*), leading to not only target alterations that reduce the binding affinity of the drugs but also the presence of plasmid-mediated quinolone resistance (PMQR) encoding genes of the *qnr* alleles [9]. These acquired antimicrobial resistance determinants are public health hazards because plasmids whose genes encode antibiotic resistance can expand such resistance through horizontal transmission [10]. For example, food animals may be consumed and their antibiotic resistance genes are transferred to the human gut microbiota [11].

This study aimed to characterize antibiotic resistance and some of its associated resistance determinants in different *S. enterica* subsp. *enterica* serovars isolated from pigs in Argentina.

## Materials and Methods

### Ethical approval

The working protocol and the used techniques comply with the regulations of the Subcommittee on Animal Bioethics under the Ethics Committee of Scientific Research, as established in Resolution 376/22 of the Superior Council of the National University of Rio Cuarto.

### Study period and location

This study was conducted from March 2018 to May 2021, at the National University of Río Cuarto, Córdoba, Argentina.

### Study population

We retrospectively analyzed antibiotic resistance and its genetic bases in strains of *Salmonella* isolated from pigs between 2011 and 2015 in the Pampean region of Argentina. We selected isolates of clinical origin (n=11), recovered from cases of lung (6), liver (1), kidney (1), and intestinal (3) lesions, and mesenteric lymph nodes (n=9). Pigs were periodically monitored at slaughterhouses and admitted to the Pathology Department Diagnosis Service. We also included 35 strains from a previous study of 1518 fecal samples collected at 52 commercial farrow-to-finish pig farms in the region, where 22 farms tested positive for *Salmonella* spp. [4]. Isolates from clinical cases were recovered using conventional bacteriology with sheep blood agar and MacConkey agar, and mesenteric lymph nodes and fecal samples were analyzed according to ISO 6579:2002 standard. All isolates were serotyped according to the ninth edition of the White-Kauffmann-Le Minor scheme (Pasteur Institute, France). The serovars of the 55 *Salmonella* strains selected were *Salmonella* Anatum (9), *Salmonella* Brandenburg (2), *Salmonella* Bredeney (1), *Salmonella* Choleraesuis (8), *Salmonella* Derby (7), *Salmonella* Glostrup (3), *Salmonella* Heidelberg (2), *Salmonella* Infantis (1), *Salmonella* Livingstone (1), *Salmonella* Montevideo (1), *Salmonella* Oranienburg (2), *Salmonella* Panama (2), *Salmonella* Rissen (3), and *Salmonella* Typhimurium (13).

### Antimicrobial susceptibility

The antimicrobial susceptibility evaluation of each strain/serovar was assessed using the Kirby-Baüer method (diffusion in Mueller-Hinton agar) according to the Clinical and Laboratory Standards Institute guidelines for the following antibiotics (n=21): Ampicillin, amoxicillin-clavulanic acid, piperacillin-tazobactam, cephalothin, cefepime, cefotaxime, cefoxitin, ceftazidime, ertapenem, imipenem, gentamicin, amikacin, azithromycin, tetracycline, ciprofloxacin (CIP), levofloxacin, nalidixic acid (NAL), trimethoprim-sulfamethoxazole, chloramphenicol, fosfomycin, and nitrofurantoin. Susceptibility breakpoint levels were those described for Enterobacterales by the Clinical and Laboratory Standards Institute [12]. A strain of *Escherichia* *coli* ATCC 25922 was incorporated to evaluate the quality of the process. A multidrug-resistant (MDR) strain was defined as non-susceptibility to at least one agent in three or more antimicrobial categories.

### Gene amplification and sequencing

The *inv*A gene was amplified to confirm all *Salmonella* isolates. Polymerase chain reaction (PCR) was performed to detect genes encoding antibiotic resistance: *Tem* to detect transferable β-lactamases, *intl*1 to detect Class 1 integron, *qnrB* to detect PMQR, and amino acid substitutions of QRDRs in *gyrA*.

All target genes were amplified using PCR with 1× DNA Taq buffer, 1 U of DNA Taq polymerase (Inbio Highway, Tandil, Buenos Aires, Argentina), 1.5 mM of MgCl_2_, 200 μM of dNTPs, 10 pmoL of each primer [13,14] (Table-1), and 2.5 μL of template DNA, for a total volume of 25 µL. DNA was physically extracted by boiling, and the PCR conditions were as follows: Incubation at 94°C for 4 min, followed by 35 cycles of 94°C for 30 s, annealing temperature according to the target gene (Table-1) for 30 s, and 72°C for 30 s, and a final extension at 72°C for 10 min.

**Table 1:** Primers, annealing temperatures, and references for each target gene.

Primer	Target gene	Sequence (5’-3’)	Reference	Annealing temperature (°C)
OT-1	*tem*	TTG GGT GCA CGA GTG GGT TA	[13]	55
OT-2		TAA TTG TTG CCG GGA AGC TA		
qnrB-F	*qnrB*	CCG ACC TGA GCG GCA CTG A	Antimicrobial Service, INEI-ANLIS “Dr. Carlos G. Malbran,” Argentina	55
qnrB-R		CGC TCC ATG AGC AAC GAT GCC T		
intI1F	*intI1*	ATC ATC GTC GTA GAG ACG TCG G	[14]	55
intI1R		GTC AAG GTT CTG GAC CAG TTG C		
gyrA-F	*gyrA Enterobacteriaceae*	ACG TAT TGG GCA AYG ACT GGA	Antimicrobial Service, INEI-ANLIS “Dr. Carlos G. Malbran,” Argentina	50
gyrA-R		CAA CGA AAT CGA CCG TCT CTT		

*GyrA* was amplified in all strains resistant to NAL. The ADN Puriprep GP-kit (Inbio Highway) was used to purify PCR products before sequencing. Amplified fragments were sequenced in both directions (5´-3´ and 3´-5´) (ABI 3130×l, Applied Biosystem, INTA Castelar, Argentina), and sequences were edited using BioEdit, aligned with ClustalW, and analyzed to detect mutations.

## Results

### Antimicrobial susceptibility

**Figure-1:**
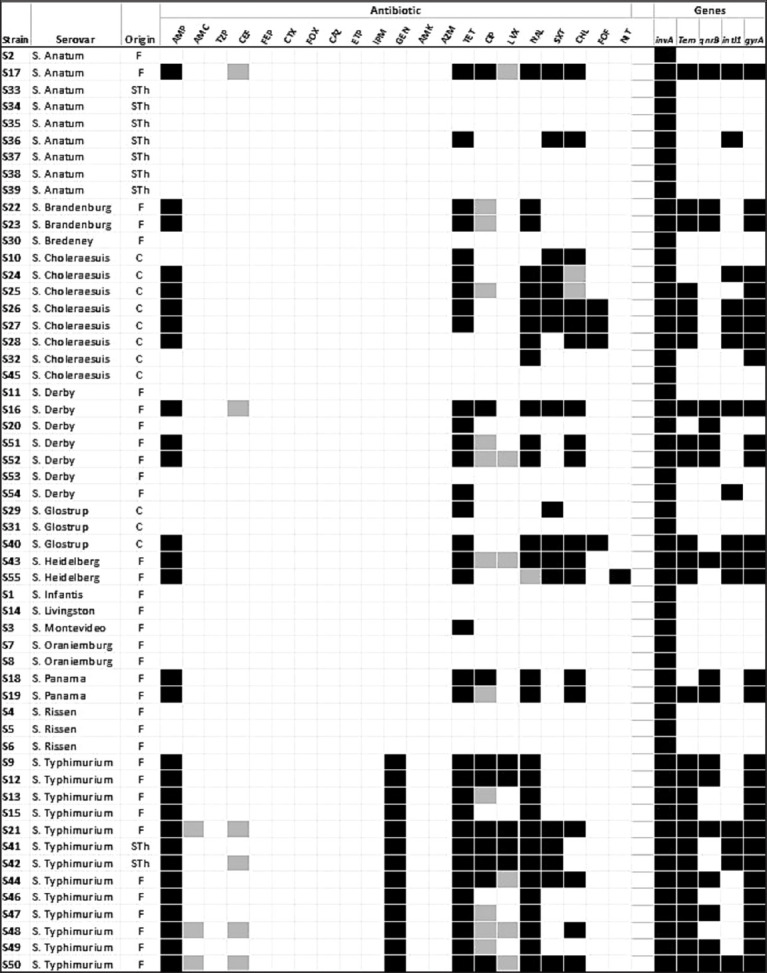
Antibiotic susceptibility of *Salmonella* strains from pig origin, showing the different categories: Black=Resistant, Gray=Susceptible with extended exposition, White=Susceptible; and also presence (black) or absence (white) of target resistance genes. The origins of the strain were field (F), slaughterhouse (Sth), and clinical cases (C).

Of the strains identified, 56% were MDR, including all strains of *S*. Typhimurium, *S*. Heidelberg, and *S*. Panama, and 75% of *S*. Choleraesuis (Figure-1). One strain of *S*. Typhimurium (S50) was resistant to seven antibiotics and showed decreased susceptibility to another three antibiotics (Figure-1). Resistance to tetracycline (62%) was most frequently observed, followed by resistance to ampicillin (52%), NAL (52%), chloramphenicol (33%), and trimethoprim-sulfamethoxazole (31%). Resistance to aminoglycosides was observed only for gentamicin in all *S*. Typhimurium strains. High susceptibility was found to β-lactams, except for ampicillin, and only one strain was resistant to fosfomycin (Figure-1).

### Antibiotic resistance genes

The gene encoding transferable β-lactamase TEM was detected in 49% of the analyzed strains and 93% of ampicillin-resistant *Salmonella*. Class 1 integrons were present in 27% of the strains, of which 80% were MDR (Figure-1). In 30 isolates resistant to NAL, 21 had a *gyrA* mutation, and 53% of NAL-resistant strains showed the *qnrB* gene. Mechanisms generated by both genes (*gyrA* and *qnrB*) were present in 76% of strains resistant to CIP. The most observed *gyrA* mutation was Ser83Tyr (12/21, 57%), followed by Ser83Phe (7/21, 33%) and Asp87Gly (1/21, 5%) in the 21 *Salmonella* strains resistant to quinolones. A double mutation Ser83Tyr+Asp72Glu was noted in one strain.

## Discussion

*Salmonella* threatens public health by causing not only clinical foodborne diseases worldwide but also extensive antibiotic resistance, which has been recently confirmed in strains isolated from various livestock species. Pork is an important protein source in Argentina, but its antibiotic resistance in the country and the underlying mechanisms of *Salmonella* isolated from pigs remain unclear. This study presents antibiotic resistance data from isolates collected from Argentinian in-farm pigs. Unlike other studies, this approach does not include *Salmonella* contamination from other sources, such as during transport to the abattoir or a stay in the lairage.

More than half of the isolates were MDR, which is consistent with the previous studies reported in other countries [15,16]. A study in Argentina found that 88% of *Salmonella* isolated from two pig farms was MDR and showed greater resistance to β-lactams than the present study [2]. However, all strains in the present study showed non-susceptibility to drugs in three or more antimicrobial categories if we consider the most virulent serovars, such as Typhimurium, which was also the most frequently isolated serovar from farms.

The strong relationship between serovar and decreased sensitivity to multiple antibiotics has been described previously [10,17]. *S*. Typhimurium is usually the most resistant serovar. Yet, our study detected strong resistance patterns in less virulent serovars such as Derby or Brandenburg. Interestingly, *S*. Panama showed consistent MDR, which was recently linked to infection of extraintestinal sites in humans, causing septicemia, meningitis, and osteomyelitis [18].

Tetracycline resistance was the most common resistance phenotype and widely distributed among *Salmonella* strains recovered from pigs, even in apparently less virulent serovars, such as *S*. Montevideo and *S*. Glostrup. Resistance to this drug has been reported from not only in this region and Brazil [19] but also worldwide [9,20]. Meanwhile, resistance to ampicillin, gentamicin, and quinolones is also consistent with reports in other countries, as shown by *Salmonella* strains isolated from clinical cases in pigs in the United States [17]. Resistance to sulfonamides has been reported worldwide [17,20]. We found lower levels here, even though high levels of resistance to sulfonamides have been reported in *Salmonella* isolated from retail market samples in Argentina [3].

Recent studies reported increasing resistance to β-lactams and cephalosporins such as ceftiofur [2,9]. Here, we found a relatively low susceptibility to ampicillin, which could be attributed to the presence of transferable β-lactamase TEM in resistant strains, yet resistance to expanded-spectrum cephalosporins was not observed. Class A β-lactamase enzymes catalyze the hydrolysis of the amide bond present in the β-lactam ring, yielding an ineffective inhibitor of the penicillin-binding proteins that target penicillins and early generation cephalosporins. The resulting high-level bacterial resistance spreads among Enterobacteriaceae, as we also found in this study [8]. These findings have significant implications for human health because this large class of antibiotics is used to treat human infections. The administration of these antibiotics has increased since most of the isolates in the present study were collected from farms more than 5 years ago. We detected extensive susceptibility to carbapenems, which agrees with other studies [20]. However, increased resistance to cephalosporins and carbapenems in the European Union in recent years warrants continued surveillance of this group of antibiotics in this region [1].

Resistance to fluoroquinolones is concerning, as quinolone-resistant strains increase in livestock production, such as CIP found in the present study and reported in Brazil, where more than 90% of the *Salmonella* isolates from pigs were resistant to CIP [19]. This class of antibiotics is also widely administered to humans, who are susceptible to many mechanisms of resistance transfer.

We analyzed QRDRs for *gyrA* mutations and found that codon 83 was the most frequent hot spot, corresponding to 90% of the mutations and all of them in NAL-resistant strains. This finding is consistent with a previous study, but we found a considerably lower proportion of codon 87 mutations [15]. The Ser83Phe mutation is frequently reported in *S*. Typhi strains, whose decreased susceptibility to CIP was detected in clinical cases in Peru [21].

Most strains with decreased susceptibility to CIP showed a combination of both *gyr*A mutations and PMQR gene *qnrB*, which together may enhance their mechanisms [15]. PMQR encoding genes of the *qnr* alleles, such as the *qnrB19* gene, are associated with the expansion of CIP-resistant *Salmonella* strains in the United States [10]. Different variants of these plasmids in Argentina were characterized from Enterobacteriaceae clinical strains isolated from clinical cases, but CIP resistance in *Salmonella* from local pigs remains poorly characterized [16]. Future molecular studies should characterize the *qnrB* genes in this subset of strains.

## Conclusion

This study detected widespread antibiotic resistance in *S. enterica* serovars isolated from Argentinian pigs. We also identified resistance determinants, such as *gyrA* mutations and the *qnrB* gene, which will guide future molecular studies. These findings highlight the need to control antibiotic use in livestock production to prevent resistance transfer to humans.

## Authors’ Contributions

AC, MG, and JP: Designed the experiment and performed the susceptibility analysis. JP, DF, and PT: Performed molecular experiments. All authors were involved in analysis of the data, and writing and reviewing of the manuscript. All authors have read and approved the final manuscript.
